# Depression and academic engagement among college students: the role of sense of security and psychological impact of COVID-19

**DOI:** 10.3389/fpubh.2023.1230142

**Published:** 2023-08-04

**Authors:** Yuxi Tang, Weiguang He

**Affiliations:** College of Social Sciences, Shenzhen University, Shenzhen, China

**Keywords:** academic engagement, college students, COVID-19, depression, psychological impact of COVID-19, sense of security

## Abstract

**Background:**

The negative consequences of depression in college students have garnered global attention, especially in relation to academic achievement during the COVID-19 pandemic, which need critical assessment.

**Aim:**

This study investigated whether a sense of security mediated the relationship between depression and academic engagement among college students during the pandemic and whether the moderating psychological impact of COVID-19 has a moderating effect on this relationship.

**Methods:**

In this cross-sectional study, we recruited 466 college students from 30 provincial-level administrative regions in China via the Internet and used established scales to measure depression, academic engagement, a sense of security, and the psychological impact of COVID-19. The mediating and moderating effects were tested using the bootstrap method.

**Results:**

Depression was found to negatively influence academic engagement, with a sense of security partially mediating this relationship. Moreover, the psychological impact of COVID-19 was shown to have a moderating effect on this mediating process.

**Conclusion:**

This study could aid in crafting pertinent strategies to mitigate the adverse effects of depression on learning amid unexpected public health crises and foster better mental health among college students.

## 1. Introduction

The COVID-19 pandemic, an unprecedented public health crisis, not only posed a grave threat to people's physical safety, but also cast a significant shadow on their mental wellbeing (1, 2). This was particularly detrimental to those already in a fragile psychological state, such as those suffering from depression, which exacerbated their condition (3). College students, who are a high-risk group for depression, faced significant challenges during the ongoing waves of the virus (4), with a rapid global rise in depressive symptoms reported among young adults, typically those with lower resilience to psychological stress (5). This deterioration in mental health is a cause of concern, and the question of how to intervene and reduce the negative psychological aftermath following such pandemic events has become a focal point of research. Analysis of depression among college students is often tied to their academic commitment, which is central to their lives (6). Academic struggles caused by depression may, in turn, impact mental health, potentially exacerbating anxiety and depressive states, thus creating a vicious cycle (7). Some college students feel hopeless and distressed, often exhibiting confusion and avoidance in their studies (8). Investigating the mechanisms by which depression affects college students' academic engagement is of paramount importance. Not only can it provide fresh insights for more effective interventions targeting student depression, but it can also improve student learning, thus laying a solid foundation for promoting students' mental health and academic progress.

Depression is an emotional disorder characterized by frequent experiences of intense feelings such as pain, emptiness, and hopelessness (9–12). It often disrupts people's mental states and leads to an array of difficulties in their studies and lives, with severe cases resulting in self-harm and other harmful consequences (13). As an unprecedented public health crisis, the pandemic caused an incalculable disruption to students' lives and studies, coupled with increased uncertainty about their future, which increased their susceptibility to depression (14). For students with a predisposition to depression or a history of illness, the pandemic undoubtedly acted as an adverse stimulus, potentially leading to heightened feelings of despair and sorrow (15). During the pandemic, home isolation measures may have confined students to a single space for extended periods, preventing them from engaging in outdoor activities, possibly heightening their feelings of repression and irritability, and triggering more frequent depressive episodes (16). With the pandemic having increased the prevalence of depression, understanding the mechanisms through which depression affects academic engagement can provide a reference for better targeted interventions. Such interventions would not only be beneficial in addressing students' academic problems, but also in promoting their mental health.

Academic engagement refers to the interest or enthusiasm that students hold toward their studies, coupled with the time and energy they dedicate to learning (17–19). Pandemic-related pressures burdened students in dealing with issues in terms of academic setbacks, lifestyle inconveniences, and future uncertainties (20). These pressures not only brought about distress among the student population, but also potentially affected students' academic engagement (21) given the unprecedented number of issues arising during the pandemic (22). For instance, some students reported increased fatigue during study sessions, whereas others experienced diminished interest in learning, even to the point of actively avoiding it (23). The interplay between mental health issues and learning problems became evident during this pandemic (24), with both a rapid increase in the number of students exhibiting symptoms of depression and a noticeable decline in their academic engagement compared with pre-pandemic levels (25).

Investigating the mechanisms affecting academic engagement and identifying the variables that could serve as mediators or moderators during the pandemic can aid in understanding the complex interplay between depression and other factors under such novel circumstances. Determining the mediating or moderating mechanisms is likely to provide a more profound theoretical understanding of depression issues faced by adolescents, especially from an educational perspective, which is likely to be beneficial for formulating effective interventions.

Some students grappled with the profound grief of losing friends and family to COVID-19, coupled with significant setbacks in their academic and personal lives (26), which could potentially exacerbate depressive states. The pandemic drastically reduced students' interest in outdoor activities and social interactions, leading to extended periods of emptiness and loneliness (27). Additionally, economic support for college students was severely affected by the pandemic, with some students' family financial circumstances deteriorating rapidly (28). In addition to dealing with boredom due to social isolation and an uncertain future, the modes of learning for these students underwent considerable changes. Online learning became the dominant mode of emergency education worldwide. However, this abrupt shift in learning modalities left many students feeling lost, thereby intensifying their anxiety (29). This situation may have led to an increased prevalence of depressive disorders, making students more susceptible to mental health issues (30), which, in turn, may have exacerbated their already difficult predicaments. Therefore, it is imperative to study depression-related issues, particularly how depression influences academic engagement, and determine appropriate interventions.

Academic engagement reflects the level and willingness of students to invest in various learning resources (31), often involving a strong desire for knowledge, proficiency in applying various effective learning strategies, and a sense of achievement in their studies. These qualities may positively contribute to mental health (32). However, during the pandemic, students' academic engagement was severely affected (33). Students faced the challenges of online, home-based, and isolated learning due to substantial changes in their learning environments (34). Discomforting feelings, including anxiety and unease, may have dampened students' enthusiasm for learning, making it difficult for them to concentrate and causing them to lose interest in their studies (35).

Depression may influence students' academic engagement through three potential pathways. During the pandemic, college students may have experienced serious psychological distress, particularly negative emotions and feelings of hopelessness triggered by depressive symptoms (36), leading to a lack of interest and an inability to gain a sense of achievement in their studies (37). The pandemic forced students to change their learning methods in a short period, and educators may have struggled to provide sufficient support through new online teaching methods, leading to potential learning burnout due to adaptation difficulties in the online learning environment (38). Some students may have significantly altered their lifestyles due to the pandemic, such as indulging in Internet use and excessively focusing on negative news about the pandemic, intensifying their negative feelings toward the pandemic (39), which could in turn have make it more difficult for them to concentrate on their studies. Therefore, it can be conjectured that college students' academic engagement may have been more influenced by depression during the pandemic.

A sense of security refers to the affirmative and positive sensations related to experiences of trustworthiness, reliability, and tranquility that arise due to one's active ability to tackle issues, have a comprehensive understanding of individuals or events, and to effectively engage with familiar environments (40–43). Among college students, insecurity is a common psychological issue (44), stemming from their lack of experience in dealing with external environments and their perceived lack of sufficient ability and resources to resolve multiple complex problems (45). Particularly during the pandemic, college students faced unprecedented events such as health threats, disruptions in their learning status, and future employment difficulties, which could have made them feel helpless (46). Under these circumstances, many students may have experienced feelings of insecurity. Among students who already had poor psychological conditions or emotional disorders, this insecurity could have potentially exacerbated their psychological issues (47), thereby severely affecting their regular learning.

During the pandemic, a sense of security among college students may have served as a mediating variable between depression and academic engagement. First, depression among college students could have potentially increased the frequency of feelings of insecurity (48). Depression is often characterized by excessive pessimism toward external matters, which can trigger worry or even panic. Moreover, long-term experiences of insecurity can negatively impact mental health, leading to increased negativity and suppression (49). Hence, there may be a strong correlation between depression and sense of security. For some students, insecurity stems mainly from the uncertainty and risks of the external environment, requiring them to expend more energy dealing with threats and risks, making it difficult to concentrate on academic challenges (50). Other students may feel insecure because of inadequacies or difficulties in their academic abilities, which may have led to potential stagnation in their learning during the pandemic, thereby exacerbating academic problems (51). These factors foster anxiety rather than enthusiasm in learning, potentially leading to a reluctance to learn. Therefore, there may be a correlation between students' sense of security and academic engagement (52). While depression in college students may have been directly linked to academic engagement during the pandemic, it may also have influenced academic engagement through feelings of insecurity, which involve distinct and intense negative emotional experiences that often directly affect students' life status and learning behavior (53). Students' depression may further amplify their feelings of insecurity, which may negatively impact their academic engagement. Based on these considerations, we inferred that a sense of security might serve as a mediator between depression and academic engagement.

The psychological impact of COVID-19 refers to psychological problems, such as distress and avoidance, caused by the pandemic (54, 55). The pandemic disrupted people's normal lives, causing some to have strong emotional reactions (56), with prolonged negative psychological effects as well as sometimes triggering anorexia, frequent nightmares, and insomnia (57). Some college students may have been prone to feelings of panic and evasion as well as a strong aversion to pandemic-related matters (58). Such increased psychological stress likely posed more challenges to their academic pursuits (59).

When the psychological impact of COVID-19 was high, college students' psychological states and normal learning may have been affected. Their mental health already faced many challenges, especially for those with depression who were struggling with emotional regulation (60). Excessive worry about the pandemic might have induced more feelings of insecurity, thereby impacting academic engagement (61). Furthermore, the psychological impact of COVID-19 may not only have potentially increased the psychological pressure on students but also affected academic engagement by diminishing learning motivation and draining energy, leading to student fatigue or a sense of futility toward studying (62). From this perspective, the psychological impact of COVID-19 may have moderated the mediating effect of a sense of security between depression and academic engagement.

Previous research has reported an association between depression and academic engagement (63, 64). While academic engagement may be adversely affected by depression directly (65, 66), it has also been reported that this relationship is contingent on specific conditions, suggesting the existence of mediating variables (67). Further studies are needed to improve understanding of the association between these factors.

Existing research has reported an association between depression and a sense of security, with individuals in depressed groups being more prone to feelings of insecurity (68–70). Academic performance is strongly correlated with student insecurity (71, 72). Individuals experiencing insecurity have been found to have their energy and interest in learning negatively affected (73, 74). Furthermore, while insecurity has been reported to mediate between psychological problems and learning (75), further investigation is required to establish whether a sense of security acted as a mediator between depression and academic engagement during the pandemic.

The psychological effects of COVID-19 could potentially have become a risk factor (76–78), possibly exacerbating adverse emotional effects (79, 80), and negatively affecting students' learning (38, 81, 82). These studies suggest that the psychological impact of COVID-19 may have served as a moderating variable.

Based on an analysis of previous related research and to help ensure better targeted interventions for depression and enhance the mental wellbeing of college students, we considered it of fundamental importance to investigate how depression affected academic engagement among college students during the COVID-19 pandemic and whether a sense of security played a mediating role under the conditions of the pandemic. Additionally, while the psychological impact of COVID-19 could potentially have acted as a moderator in this mediating relationship, this area remains relatively unexplored in existing research; therefore, we also investigated this factor. The three hypotheses of this study are as follows:

Hypothesis 1. Depression can negatively predict college students' academic engagement.
Hypothesis 2. A sense of security in college students mediated the relationship between depression and academic engagement.
Hypothesis 3. The psychological impact of COVID-19 moderated the relationship between depression and academic engagement.

**Figure 1:**
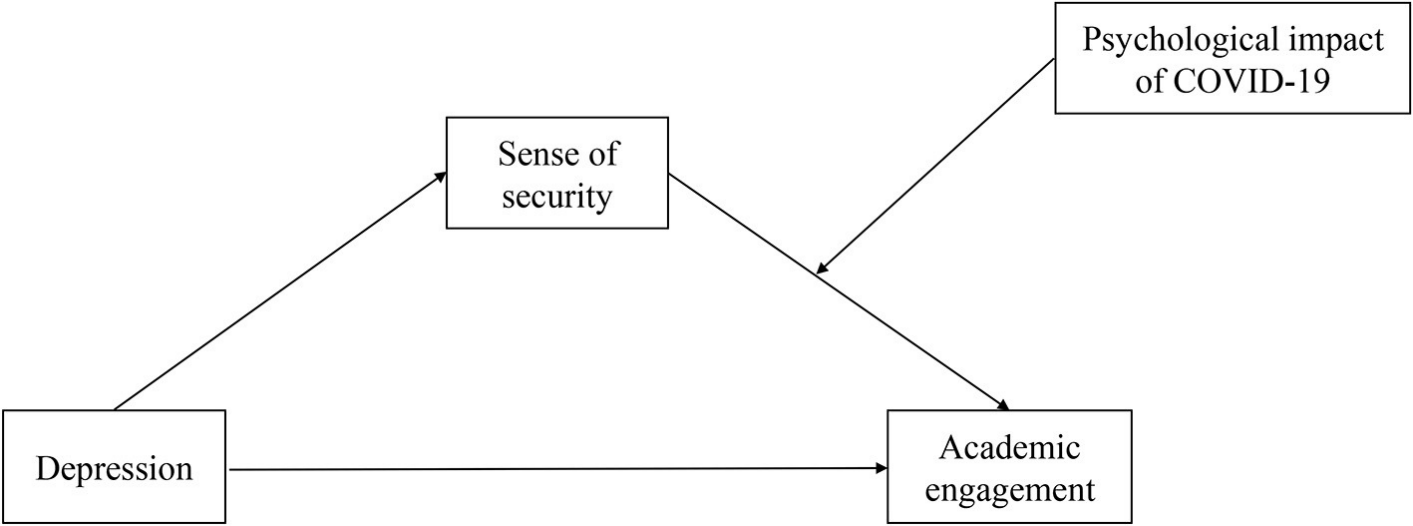
Research hypothesis model diagram.

The research hypothesis model diagram is shown in Figure 1.

## 2. Methods

### 2.1. Process and participants of the survey

#### 2.1.1. Design

This study focused on college students aged 18 years and older studying in China. This study adopted a cross-sectional design to assess the current state and associations of variables in a single time point. The sample was composed of currently enrolled, full-time college students from diverse academic disciplines across China. Inclusion criteria were: being a college student, being 18 years of age or older, currently residing in China, and being willing to participate in an online survey. Exclusion criteria included: students who were not currently enrolled, those under the age of 18, those who did not currently reside in China, and those who did not provide informed consent to participate. However, during the pandemic period in China, it was challenging to conduct offline surveys of college students from different regions across the country. Convenience sampling was therefore adopted. College students were recruited using various online platforms. College students viewed recruitment information online and voluntarily participated in the survey, with participation covering multiple regions.

#### 2.1.2. Procedure

The survey was conducted in December 2022 when normal life was affected in China due to the ongoing COVID-19 pandemic. Prior to conducting the survey, our institution conducted an ethical review and approved the study. A professional online questionnaire research platform was used to recruit college students from 30 provinces across China. We provided the participants with informed consent forms and participation was dependent on these being completed. A total of 471 people completed the questionnaire, five of whom were excluded because their response time was <3 min, and 466 questionnaires were retained. The questionnaire included four scales and collected basic demographic information. Monte Carlo analysis revealed that the sample size needed to exceed 232 for 0.8 statistical power (83). Therefore, the sample size was adequate.

#### 2.1.3. Sample characteristics

Of the final 466 questionnaire respondents included in this study, 355 were female, 111 were male, with ages ranging from 18 to 32 years (average, 21.1 ± 1.97 years), and comprising 381 undergraduates (81.76%), 76 master's students (16.31%), and 9 doctoral students (1.93%).

### 2.2. Measurement

Depression was assessed using a scale developed by Spitzer et al. (84). The Chinese version of this scale has proven reliable (85). A 4-point Likert scale is used to score each of the nine items in the scale, such as “Over the past 2 weeks, have you experienced a loss of appetite or overeating?,” where a higher total score indicates a greater level of depression. In this study, the Cronbach's α coefficient was 0.85, the McDonald's omega coefficient was 0.885, and the Kaiser-Meyer-Olkin (KMO) coefficient was 0.885.

Academic engagement was evaluated using a scale developed by Schaufeli et al. (86). The reliability of the Chinese version of this scale has been verified (87). This scale includes nine items, such as “Does your study inspire you?,” rated on a 7-point Likert scale, with a higher score reflecting higher levels of academic engagement. In this study, the Cronbach's α coefficient was 0.93, the McDonald's omega coefficient was 0.945, and the KMO coefficient was 0.932.

A sense of security was measured using a scale developed by Cong and An (87). The reliability of this scale in its Chinese version has been verified (88). The scale consists of 16 items, for instance, “Do you often feel unlucky?,” scored on a 4-point Likert scale, where a higher cumulative score signifies a stronger sense of security. The Cronbach's alpha, McDonald's omega, and KMO coefficients for this study were 0.881, 0.901, and 0.866, respectively.

The psychological impact of COVID-19 was measured using a scale developed by Vanaken et al. (89). The reliability of this scale has been verified (90). This scale includes 15 items, such as “Have you had dreams about the pandemic in the past week?,” rated on a 5-point Likert scale, with the interpretation being that a higher total score indicates a greater psychological impact of COVID-19. The Cronbach's alpha, McDonald's omega, and KMO coefficients for this study were 0.872, 0.894, and 0.898, respectively. For the questionnaire used in this study, we computed the composite reliability (CR) index, which came out to be 0.875, indicating a high degree of validity. We also carried out a confirmatory factor analysis, and found that the Parsimony Goodness of Fit Index (PGFI) was 0.586, the Parsimony Normed Fit Index (PNFI) was 0.625, the Parsimony Comparative Fit Index (PCFI) was 0.645, and the Standardized Root Mean Square Residual (SRMR) was 0.091. These model fit indices reflect a good overall fit of the model.

### 2.3. Data analysis

The scores of the college students on the four scales of academic engagement, depression, sense of security, and the psychological impact of COVID-19 were tallied, and their means (*M*) and standard deviations (*SD*) were calculated. Pearson's correlation coefficients were used to analyze the correlations. Given that the sample size was >300, kurtosis and skewness values were used to estimate the multivariate normal distribution. To test the moderated mediation effect, we used Model 14 of the microprocess plugin developed by Hayes (91) for the mediation analysis and moderation effect. A moderated mediation effect can be considered when the bootstrap confidence interval excludes zero.

## 3. Results

A normality test was conducted on the data of the four variables: academic engagement, depression, a sense of security, and the psychological impact of COVID-19. The skewness values were 0.426, −0.166, −0.008, and 0.170, respectively, and the kurtosis values were −0.326, −0.523, −0.162, and −0.134 respectively, indicating that all the variables conformed to a normal distribution. In addition to the skewness and kurtosis assessments, visual inspection of the histograms also confirmed the normal distribution, further substantiating that all four variables exhibited essential normality. Analysis of the correlations among the four variables revealed a negative correlation between depression and academic engagement (r = −0.457, *p* < 0.01) and a negative correlation with a sense of security (r = −0.258, *p* < 0.01). A significant correlation was also found between a sense of security and academic engagement (r = 0.297, *p* < 0.01). The specifications are listed in Table 1.

**Table 1:** Correlation analysis of the four variables.

	**Mean**	**Standard deviation**	**Depression**	**Learning engagement**	**Sense of security**	**Psychological impact of COVID-19**
Depression	18.779	5.32	1			
Academic engagement	37.277	10.571	−0.457^**^	1		
Sense of security	48.867	11.688	−0.258^**^	0.297^**^	1	
Psychological impact of COVID-19	34.594	8.183	0.403^**^	0.028	−0.132^**^	1

** *p* < 0.01.

**Figure 2:**
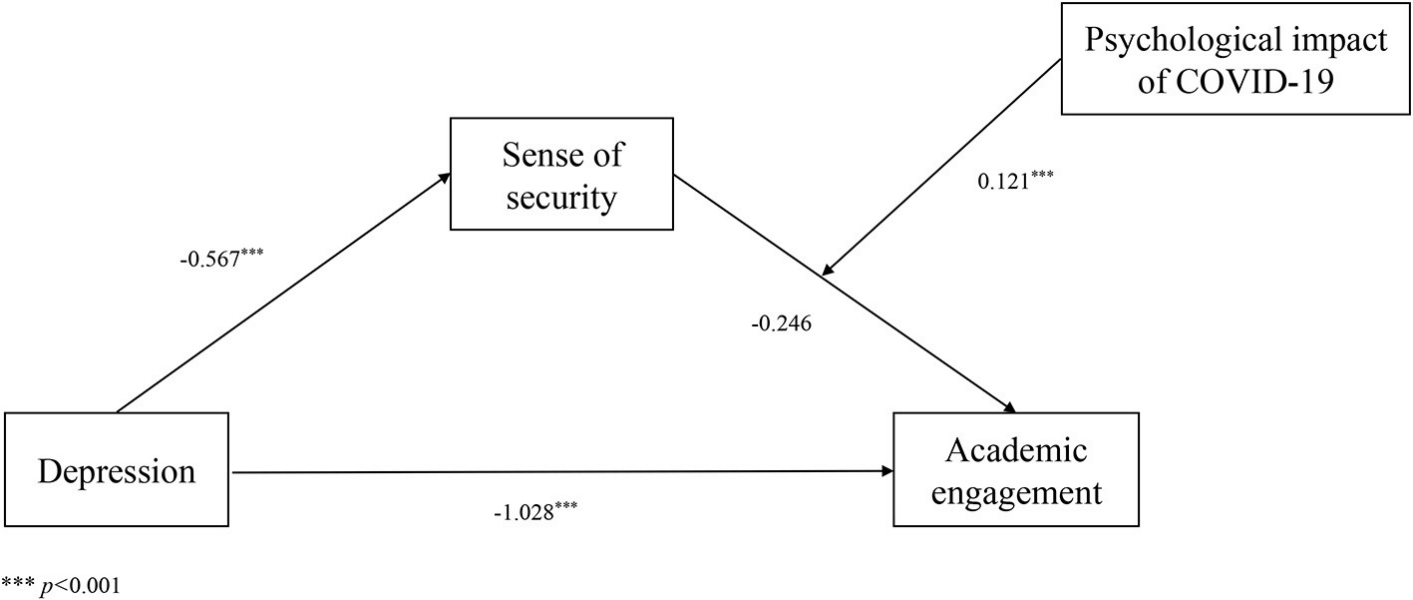
Test results of the mediator model with moderation. ^***^p < 0.001.

Regression analysis showed that depression could significantly negatively affect a sense of security (B = −0.5669, t = −5.7535, *p* < 0.001). A sense of security (B = 0.1741, t = 0.0379, *p* < 0.001) and depression (B = −0.8084, t = −9.7212, *p* < 0.001) significantly affected academic engagement (R^2^ = 0.2430, F = 74.3092, *p* < 0.001). These results suggest that depression negatively affected academic engagement and that this relationship was mediated by a sense of security. Furthermore, depression (B = −1.028, t = −11.969, *p* < 0.001), a sense of security (B = −0.246, t = −1.893, *p* = 0.059), the psychological impact of COVID-19 (B = −0.267, t = −1.446, *p* = 0.149), and the interaction between a sense of security and the psychological impact of COVID-19 (B = 0.012, t = 3.419, *p* < 0.001) significantly affected academic engagement (R^2^ = 0.317, F = 53.4556, *p* < 0.001). The test results of the mediated model with moderation proposed in this study are shown in Figure 2. In this model, the interaction between a sense of security and the psychological impact of COVID-19 was significant (95% confidence interval [CI] = [0.0051,0.0191]), indicating that a sense of security and academic engagement were moderated by the psychological impact of COVID-19 (See Table 2).

**Table 2:** Summary of regression models.

	**Academic engagement**				**Sense of security**			
	**β**	**SE**	***t***	***p***	**β**	**SE**	***t***	***p***
Constant	57.549	6.995	8.228	*p* < 0.001	59.513	1.923	30.948	*p* < 0.001
Depression	−1.028	0.086	−11.969	*p* < 0.001	−0.567	0.099	−5.754	*p* < 0.001
Impact of COVID-19	−0.267	0.184	−1.446	*p* = 0.149				
Sense of security	−0.246	0.13	−1.893	*p* = 0.059				
Sense of security × Psychological impact of COVID-19	0.012	0.004	3.419	*p* = 0.001				
R ^2^	0.317				0.067			
F	F = 53.456, *p* < 0.001				F = 33.103, *p* < 0.001			

When analyzing the moderating effect of the psychological impact of COVID-19, it was found that at a low level of impact (*M –* 1*SD*) with an effect value of −0.042 and 95%CI = [−0.110,0.010], there was no mediation. However, the mediation was significant at both *M* and high *M* levels (*M* + 1*SD*) in relation to the psychological impact of COVID-19, with effect values of −0.098 and −0.154, and 95%CIs of [−0.168, −0.042] and [−0.251, −0.068], respectively (See Table 3). Therefore, the mediating role of a sense of security varied at different levels in terms of the psychological impact of COVID-19, indicating moderated mediation.

**Table 3:** Results of the conditional indirect effect.

**Mediating variable**	**Level**	**Level values**	**Effect**	**BootSE**	**BootLLCI**	**BootULCI**
Sense of security	Low level (M – 1SD)	26.412	−0.042	0.031	−0.11	0.01
	Mean	34.594	−0.098	0.033	−0.168	−0.042
	High level (M + 1SD)	42.777	−0.154	0.047	−0.251	−0.068

**Figure 3:**
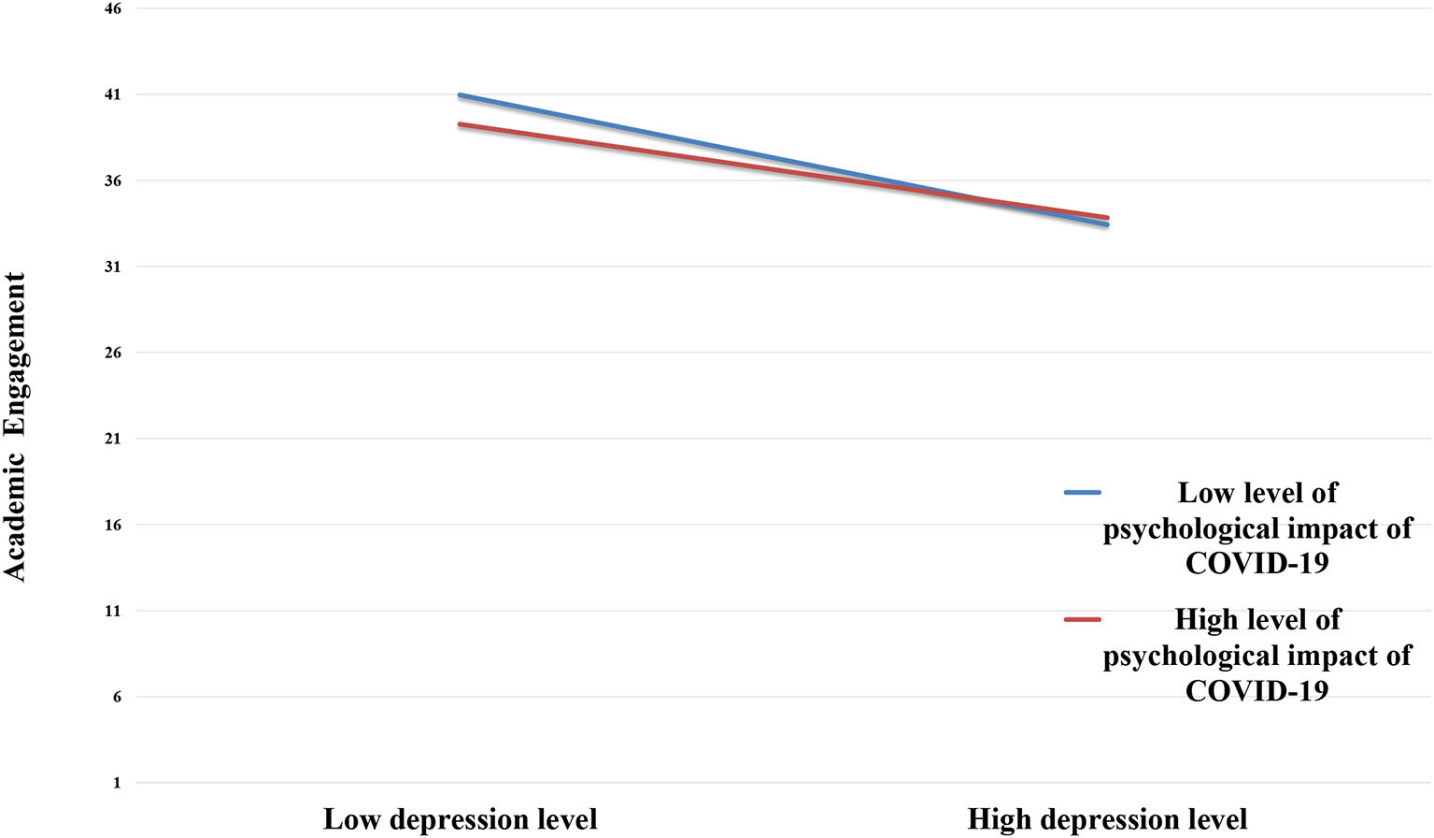
Mediating moderating effect between depression and academic engagement.

Simple slope analysis revealed that under high levels of the psychological impact of COVID-19, as the level of depression increased, the level of academic engagement was noticeably poorer compared to the group with low levels (See Figure 3). This finding indicates that the psychological impact of COVID-19 significantly moderated the mediating role of depression and academic engagement.

## 4. Discussion

This study found that depression negatively influenced college students' academic engagement during the COVID-19 pandemic, with a sense of security playing a mediating role and the psychological impact of COVID-19 having a moderating effect. As a result of these findings, hypothesis 1 and hypothesis 3 were both supported, and hypothesis 2 was partially supported. Notably, theoretical understanding of how depression affects college students in the context of sudden public health crises has been strengthened by this new insight into the relationship between depression and academic engagement.

This study found that college students' depression was negatively associated with academic engagement, supporting the first hypothesis, which accords with previous studies reporting that college students' depression affects their academic engagement (92, 93). However, in this study conducted during the pandemic, college students' depression was found to have a stronger effect on academic engagement. This finding implies that, in the context of new environmental variables, the risk factors for college students' depression affecting academic engagement are likely to be exacerbated, such as psychological difficulties, academic challenges, and financial issues, which could intensify this relationship (94).

Several factors may explain why depression negatively affects college students' academic engagement. First, depression severely affects students' psychological and emotional states (95). This emotional instability and the frequent experience of low mood can compromise learning effectiveness (35) and significantly undermine academic self-efficacy in the long run (96). Second, an enthusiastic attitude toward learning is crucial for academic engagement (97). Depression can lead to a loss of interest in learning and doubts about its importance, especially during crises such as the pandemic when students faced an uncertain future, further diminishing their enthusiasm for learning (98). Additionally, students had to deal with sudden shifts to online learning, prolonged Internet usage, and an inability to consult teachers face-to-face. Among those with depression, these substantial learning challenges and unprecedented pressures could have become overwhelming, possibly leading to avoidance or refusal to learn (99). Depression among college students during the pandemic could have intensified, significantly affecting their sleep quality and mental states and making it difficult for them to concentrate on academic issues (100). Therefore, students' academic engagement was more likely to be significantly affected by depression, with the pandemic having a further exacerbating effect.

This study found that under certain levels of psychological impact of COVID-19, a sense of security partially mediated the relationship between depression and academic engagement, partially confirming the second hypothesis. Previous research has suggested that depression may influence feelings of insecurity (101), which aligns with the results of the present study. This study also found a correlation between college students' sense of security and academic engagement, further corroborating previous studies (102, 103).

Symptoms of depression in college students usually form over a long period owing to the combined influence of various factors, and their impact on students' learning and life may require other elements to unfold further (104, 105). Depression in college students is often characterized by significant emotional issues (106), which alter their perceptions of and attitudes toward their surroundings. During the pandemic, students exhibiting depressive symptoms were more likely to worry about their personal safety and future prospects (107), which negatively affected their passion for learning. Students' heightened sense of insecurity during the pandemic indicates that they faced challenges in terms of adapting and problem-solving, which may have disrupted their learning mindset. In the complex pandemic environment, college students required a calm and stable mindset for academic engagement (108). The greater insecurity induced by depression imposed enormous pressure on some of them, preventing them from focusing on their studies (109). Importantly, depression makes students more susceptible to perceived threats. When students are highly anxious and fearful, learning is not a priority in their subconscious, especially during crises such as the pandemic when they had to divide their attention (110). Consequently, the psychological resources that can be allocated to learning naturally decrease, thereby affecting academic engagement.

This study found that depression and academic engagement in college students were moderated by the psychological impact of COVID-19, thereby verifying the third hypothesis. There was a greater likelihood that depression would affect academic engagement among those who experienced a high level of psychological impact from COVID-19, supporting previous findings (111–114). This finding suggests that in college students with depression, sudden major public health crises may further disrupt mental states; therefore, along with routine depression interventions, it is crucial to address heightened emotional stress due to crises such as the pandemic.

There are several key reasons why the psychological impact of COVID-19 mediated the relationship between depression and academic engagement. First, the psychological impact of COVID-19 involved short-term and intense negative emotions (115). These types of emotion could have amplified negative feelings among depressed college students, causing them to experience more anxiety and pressure, which depleted their energy for learning. Second, among those experiencing strong pandemic-related stress, their ability to regulate emotions may have been significantly affected (116), with emotional dysregulation becoming more severe. This outcome could have weakened their ability to manage their feelings of insecurity, thereby reducing their enthusiasm for learning. Good cognitive abilities are necessary for academic engagement. In groups that reacted excessively to the pandemic, their cognitive abilities might have been further weakened (117), thus exacerbating the negative influence of depression on academic engagement.

### 4.1. Main contributions

This study provides novel theoretical insights. First, it was found that college students' sense of security partially mediated the relationship between depression and academic engagement. The degree to which COVID-19 impacted students psychologically was also found to moderate this mediating effect. This study offers a fresh perspective on the mechanisms underlying the interaction between depression and learning among college students. Second, the analysis of the psychological impact of COVID-19 provides new evidence for understanding depression-related issues during major public health crises. Finally, this study enhances understanding of the mechanisms underlying the negative effects of depression, which can help generate new ideas for the development of better targeted intervention strategies.

### 4.2. Practical implications

In accordance with the findings of this study, during critical events such as the COVID-19 pandemic, the mental health support for college students should be brought to the forefront. It has been observed that during such severe public health crises, not only does the prevalence of depression among college students significantly rise, but mental health of already depressed individuals is further impacted. Hence, a concerted effort to educate college students about their mental health, as well as the provision of timely psychological counseling support, becomes vital. Firstly, in the face of the pandemic, institutions should ramp up their psychological aid services to assist students in handling the mental pressures triggered by the crisis. This would include proffering counseling and targeted assistance, incorporating both individual and group psychological therapies. Secondly, educational institutions should proactively disseminate pandemic-centric mental health education. This can take the form of online mental health seminars, aimed at enlightening students about the psychological repercussions of the pandemic, equipping them with methods to assuage the mental stress incited by the situation, and instructing them on preserving their academic engagement amidst these arduous circumstances. Additionally, fostering tighter collaboration between an institution's mental health services department and its basic academic units can be beneficial. This partnership, expressed through the organization of relevant activities, can boost students' psychological resilience, enhance their mental health literacy, and arm them with the tactics to effectively navigate through similar crises. Moreover, the institutions bear the responsibility of ensuring a secure environment for the students' living and learning needs. For those students whose in-person learning has been obstructed due to the pandemic, schools should guarantee a safe, stable online platform to ensure the seamless continuation of their studies. It would also be advantageous to schedule frequent online social events, allowing students to experience the warmth and support of their community, even while confined at home. Lastly, and most importantly, institutions need to create an exhaustive protocol to manage student mental health crises during public health emergencies. This could entail setting up mental health records, offering active interventions for students grappling with depression during ordinary times, and emphasizing the provision of psychological counseling services, online learning resources, and flexible academic policies in the event of sudden public health emergencies. In doing so, institutions can secure students' sense of safety and academic involvement during crises, thereby mitigating the potential risks associated with depression. This study uncovered that the academic engagement of college students was severely challenged during the pandemic. This necessitates focused interventions to ensure these students receive additional attention and assistance, fostering a supportive learning environment that diminishes feelings of insecurity and encourages active academic involvement. It underlines that during unexpected public health crises, students should be offered counseling and specific assistance to address their psychological needs. Overall, these steps can significantly contribute to the mitigation of adverse effects on learning due to depression and foster better mental health among college students.

### 4.3. Limitations

This study had some limitations. The data collected in the research were derived from self-reporting, which may have resulted in bias as students may have avoided providing truthful responses due to perceived social conformity constraints. Future studies should employ multiple data collection methods to obtain more comprehensive and accurate datasets. All participants in this study were from China and, because of cultural differences, the findings may not be generalizable to other cultures. Further validation of these findings in other countries is warranted. This study used convenience sampling. Although a wide range of students from different regions was recruited online to increase the breadth of the sample, future studies should employ more systematic sampling methods for a more accurate representation of Chinese college students. Female participants constituted a large proportion of the investigation, which might have introduced some bias. Future studies could control for the gender ratio of the participants. In addition to a sense of security and the psychological impact of COVID-19, other factors and mechanisms may have been involved in terms of the relationship between depression on student engagement, which should be explored further in future studies. In addition, the investigation was cross-sectional; therefore, causal inferences could not be made, suggesting the need for experimental or longitudinal research designs in future investigations.

## 5. Conclusion

This study revealed that during the COVID-19 pandemic, depression among college students negatively affected their academic engagement. A sense of security partially mediated this relationship, with the psychological impact of COVID-19 found to moderate this mediating effect. Furthermore, the indirect negative effect of depression on academic engagement through a sense of security was found to be stronger in students who experienced a higher psychological impact from COVID-19. These findings provide new insights into the mechanisms through which depression affects academic engagement among college students and new evidence of the negative impact of depression on students. This study can help inform the development of more effective intervention strategies during crisis events such as the pandemic that can reduce the negative effects of depression and promote students' academic engagement.

## Data availability statement

The raw data supporting the conclusions of this article will be made available by the authors, without undue reservation.

## Ethics statement

The studies involving human participants were reviewed and approved by Academic Committee of Shenzhen University's College of Social Sciences. The patients/participants provided their written informed consent to participate in this study.

## Author contributions

YT responsible for the execution of the methodology, securing the required funding, and drafting the original document. WH conducts formal analysis, provides review and editing of the manuscript, and manages the project. The investigation aspect has seen contributions from both YT and WH.
